# Overproduction of Glucose Oxidase by *Aspergillus tubingensis* CTM 507 Randomly Obtained Mutants and Study of Its Insecticidal Activity against *Ephestia kuehniella*

**DOI:** 10.1155/2020/9716581

**Published:** 2020-06-05

**Authors:** Mouna Kriaa, Hanen Boukedi, Marwa Ben Rhouma, Yosri Ben Nasr, Slim Tounsi, Lotfi Mellouli, Radhouane Kammoun

**Affiliations:** ^1^Laboratory of Microorganisms and Biomolecules, Centre of Biotechnology of Sfax, University of Sfax, P.O. Box “1177”, 3018 Sfax, Tunisia; ^2^Laboratory of Biopesticides, Centre of Biotechnology of Sfax, P.O. Box “1177”, 3018 Sfax, Tunisia; ^3^Higher Institute of Biotechnology of Sfax, B.P. 261, Sfax 3000, Tunisia

## Abstract

In order to enhance the production of glucose oxidase (GOD), random mutagenesis of *Aspergillus tubingensis* CTM 507 was performed using the chemical and physical mutagens: nitric acid and UV irradiation, respectively. The majority of the isolated mutants showed good GOD production, but only some mutants presented a significant overproduction, as compared with the parent strain. The selected mutants (19 strains), showing an overproduction larger than 200%, are quite stable after three successive subcultures. Among these, six strains revealed an important improvement in submerged fermentation. The insecticidal activity of GOD produced by the wild and the selected mutant strains was evaluated against the third larval instars of *E*. *kuehniella*. Mutant strains U11, U12, U20, and U21, presenting the most important effect, displayed an LC_50_ value of 89.00, 88.51, 80.00, and 86.00 U/cm^2^, respectively, which was 1.5-fold more important than the wild strain (61 U/cm^2^). According to histopathology observations, the GOD enzyme showed approximately similar damage on the *E*. *kuehniella* midgut including rupture and disintegration of the epithelial layer and cellular vacuolization. The data supports, for the first time, the use of GOD as a pest control agent against *E*. *kuehniella*.

## 1. Introduction

The nucleopolyhedrovirus from the Mediterranean flour moth, *Ephestia kuehniella* (Lepidoptera: Pyralidae), known also as mill moth, is a worldwide pest which causes considerable damage to stored grains, legumes, dried fruits, dates, and other stored food [1, 2]. Its larvae reduce product quality by the production of frass and webbing and cause direct damage by feeding. In Tunisia, *E*. *kuehniella* is considered a major pest of many stored food [3]. Over the past decades, several approaches have been made to control this pest through the development of chemical insecticides [4, 5]. However, the extensive use of these chemicals is not always efficient to control insect pest species, owing to the emergence of insect resistance [3, 6, 7]. As an alternative to chemical insecticides, various microorganisms such as *B*. *thuringiensis* [2, 8, 9] have been found to be effective against *E*. *kuehniella*. Since there are few described biological agents active against *E*. *kuehniella* [3, 10], we propose to test the glucose oxidase (GOD), a new bioactive agent with great insecticide potential against *E*. *kuehniella*. In fact, the success of this enzyme as a potential biocontrol agent of tomato plant diseases caused by *Fusarium solani* [11] and its antifungal efficiency against *Pythium ultimum* [12] led to the investigation of this new biological agent as an alternative to chemical insecticides.

Glucose oxidase (*β*-D-glucose: oxygen-oxidoreductase EC 1.1.3.4) is a glycoprotein that belongs to the oxidoreductase family. This enzyme catalyzes the oxidation of *β*-D-glucose into hydrogen peroxide and gluconic acid using molecular oxygen as an electron acceptor. GOD has several biotechnological applications. It has been widely used as a potent antibacterial and antifungal agent in food industry and in agriculture [11, 12]. Several studies reported that GOD from the labial gland of herbivorous insects suppressed infectivity of potential pathogens. It has the capacity to beat a broader range of insect pathogens [13, 14]. Through its catalytic product hydrogen peroxide, the labial gland GOD importantly inhibit the direct and indirect plant defenses by attenuating the ethylene and jasmonic acid levels and eliciting the salicylic acid burst [14, 15]. It was demonstrated that GOD from *H*. *zea* saliva and *Ostrinia nubilalis* induced the defense responses in tomato [16]. To the best of our knowledge, no study has been conducted, so far, to evaluate the insecticidal activity of the microbial GOD. Accordingly, in this study, we aimed to enhance *Aspergillus tubingensis* CTM 507 glucose oxidase production through random mutagenesis using the chemical (nitric acid) and physical (UV irradiation) mutagens. We investigated also the insecticidal activity of the partially purified wild and mutant GOD against the third larval instars of *E*. *kuehniella*. Indeed, we focus to develop a new bioinsecticide that could be used as a natural, cost-effective, and potent alternative to control pests on plants.

## 2. Materials and Methods

### 2.1. Microorganism

The microorganism used in this study was previously isolated from a contaminated cereal sample and was identified as *Aspergillus tubingensis* CTM 507 [11].

The fungus was inoculated onto a potato dextrose agar (PDA) medium plate at 30°C. The spores on the PDA 48-hour-old culture were harvested, counted microscopically (2.10^7^ spores/ml), and served as inocula.

### 2.2. Media

*Screening medium* used for the preliminary selection of GOD-producing mutants contains 10 g/l of glucose; 6 g/l of NaNO_3_; 0.5 g/l of KCl; 0.5 g/l of MgSO_4_·7H_2_O; 1.5 g/l of KH_2_PO_4_ with traces of CuSO_4_, ZnSO_4_, MnCl_2_, and FeSO_4_; 20 g of agar; and 2.5 mM o-dianisidine [17].

*Liquid medium* used for the second selection of the overproduced GOD mutants contains 20 g/l sucrose, 4 g/l yeast extract, 2 g/l corn steep liquor, 2 g/l peptone, 0.5 g/l KCl, 0.5 g/l MgSO_4_·7H_2_O, and 1.5 g/l KH_2_PO_4_ with traces of CuSO_4_, ZnSO_4_, MnCl_2_, and FeSO_4_ [11].

### 2.3. Mutagenesis

#### 2.3.1. Spore Treatment with UV Irradiation

The mutagenesis was performed by UV irradiation treatment of the spore suspension (2.10^7^ spores/ml). The inoculum was UV irradiated for various exposure time intervals (5, 10, 15, 20, and 25 min) using a germicidal lamp (UV Lamp: Type A-409, P.W. Allen and Co., 253-Liverpool, RD., London N.1) emitting light at a wavelength of 254 nm at a distance of 15 cm and kept for 2 hours in the dark for the stabilization of thymine-thymine dimers. For each exposure time, 100-fold serial dilutions of spores with mutation were prepared and 0.1 ml of a diluted volume was spread onto PDA media.

#### 2.3.2. Spore Treatment with Nitric Acid

*Aspergillus tubingensis* CTM 507 was also subjected to chemical mutagenesis using nitric acid as mutagen at the final concentration of 0.3 mg/ml. One ml of nitrous acid solution and 9 ml of spore suspension of *A*. *tubingensis* (2.10^7^ spores/ml) were added in a flask and kept in a water bath (30°C) for different durations (5 to 120 min). After treatment, 1 ml sample was drawn and washed twice with K_2_HPO_4_ solution (0.2 M) and bidistilled water. Serial dilutions were used to calculate the lethality percentage as described above.

#### 2.3.3. Selection of Mutants

The first selection of GOD-active mutants was done on the cell viability. After incubation at 37°C for 24 hours, the survival colonies were counted and the dose survival curve was plotted for time of mutagenesis agent exposure against the percentage of survival.

Percentages of lethality were calculated as follows:

(1) $$\begin{array}{c} \text{Lethality yield }\left(\%\right)=100-\frac{N_{\text{t}}}{N_{0}}\times 100, \end{array}$$

where *N*_t_ is the number of viable spores of treated suspension and *N*_0_ is the number of viable spores of untreated suspension.

The plates showing 99% survivors were further screened for glucose oxidase activity. Hyperproducing GOD mutants were secondly identified on an agar plate containing 0.1 g/l o-dianisidine and 20 mg/ml of horseradish peroxidase (HRP) giving rise to a brown color. The diameter of the brownish-red halos around the fungi (*D*) and the diameter of the fungal growth (*d*) were measured. The mutants with major *D*/*d* ratios were selected for further studies.

Efficiency of the selected mutants to produce GOD was then evaluated more accurately by cultivation at the optimized conditions in submerged and solid-state fermentation.

### 2.4. Glucose Oxidase Preparation

Following 32 hours of cultivation in the optimized medium, the extracellular GOD enzyme was separated from the culture medium by filtration and centrifugation. The enzyme preparation was then partially purified by heat treatment (10 min at 50°C) and 70% ammonium sulfate fractional steps [11].

### 2.5. Enzyme Assay

Glucose oxidase activity was assayed in a reaction mixture (3.1 ml) containing 0.1 ml of the enzyme and 2 ml of 1 M glucose solution prepared in sodium acetate buffer (0.1 M, pH 5.0). The reaction mixture was incubated at 35°C for 10 min. The hydroquinone liberated in the reaction mixture was measured according to the fast spectrometric method at 290 nm [11].

### 2.6. Bioassays

Bioassays were carried out using the first instar (L1) larvae of *Ephestia kuehniella* under starvation for 20 h. Partially purified GOD from the wild and mutant strains was tested at different concentrations (20, 40, 60, 80, and 100 U/ml), and each test was done in triplicate. Ten *E*. *kuehniella* larvae were placed in a sterile Petri dish containing 1 g of semolina mixed with the GOD extract. As the negative control, larvae were fed with semolina treated by buffer solution. The plates were incubated for 6 days in the insect culture room under controlled conditions of temperature of 23°C, relative humidity of 65%, and a photoperiod of 18 h light and 6 h dark. Mortality was recorded up to 3 days, and fifty percent lethal concentration (LC) was calculated from probed raw data by probit analysis using programs written in the R language [18].

### 2.7. Histopathological Effect of GOD in the Midgut of *Ephestia kuehniella*

After 6 days of exposure to GOD, *E*. *kuehniella* larvae were fixed in formaldehyde buffer solution (10%) at 4°C. Samples were dehydrated in increasing ethanol concentrations, washed by toluene (100%), and then impregnated and embedded in paraffin wax. Ultrathin sections (5 ml) were placed in carriers loaded with a mix of 15 egg albumin and 3% glycerol in distilled water. After that, sections already deparaffinized in 100% toluene were stained with hematoxylin-eosin as described by Ruiz et al. [19]. Images were observed and photographed using a light microscope (Olympus Optical Co. Ltd.) operating at an Olympus DP70 camera.

### 2.8. Statistical Analysis

All the experiences were replicated in triplicate, and the results are mean and standard deviation (±SD) of the value.

Data were analyzed using SPSS (Version 11.0.1 2001, LEAD Technologies, Inc., USA) statistical software. Mean values among treatment were compared using Duncan's multiple range test at the 5% (*P* ≤ 0.05) level of significance.

## 3. Results and Discussion

### 3.1. Effect of UV Rays and Nitric Acid on Cell Viability

**Figure 1:**
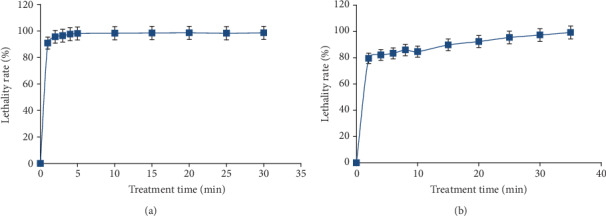
Lethality rate for *A*. *tubingensis* CTM 507 after nitrous acid (a) and UV irradiation (b) treatment for different time intervals.

The wild strain *A*. *tubingensis* CTM 507 was irradiated by UV rays or treated with nitric acid at different times (1 to 35 min) to improve its productivity. The obtained results showed that the wild strain was sensitive to the treatments. Indeed, high frequencies of lethality were reached with acid treatments and UV exposition. This result was expected since various studies demonstrated that UV radiations produce thymine via the deamination of 5-methylcytosine resulting in a G-C to A-T transition [20]. However, the effect of nitrous acid on nucleic acids causes the deamination of the amino groups of the adenine and gives rise to A-T → G-C transition [21]. The finding revealed also that the survival rate of mutants was severely affected by the time exposure. The treated culture of *A*. *tubingensis* CTM 507, with nitric acid at a concentration of 0.3 mg/ml, showed an important lethality rate of 90% after one minute of exposure. It was gradually increasing with exposure time to reach 100% after 5 min (Figure 1(a)). Similarly, UV ray treatment (*λ* = 254 nm) generates a high rate of lethality, 80% during the first two minutes. The lethal action of this mutagen is greatly increased causing 90% and 99% of mortality after 15 and 35 min, respectively (Figure 1(b)). The decrease in survivability with the increase in exposure time was reported by some other studies [22, 23]. These reports noted that the survivability of the parent strain depended on the nature of the microorganism, the type of mutagens, and the treatment period.

### 3.2. Selection of Overproductive Mutants

The survival rate is a good indication of the effectiveness of mutagenic treatment when it varies between 1 and 5%. The high frequencies of mutation lead to a small number of survivors with high frequencies of overproducing mutants. Bapiraju et al. [24] reported 99% killing and less than 1% survival for the spores of *Rhizopus* sp., for the enhanced production of lipase. In this study, the first selection of the mutants was based on the survival rate of 1%.

**Figure 2:**
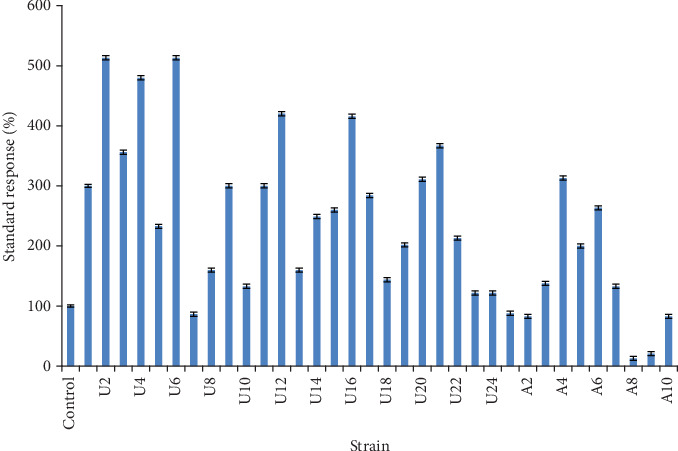
Standard response of the fungus *A*. *tubingensis* and its mutants.

**Figure 3:**
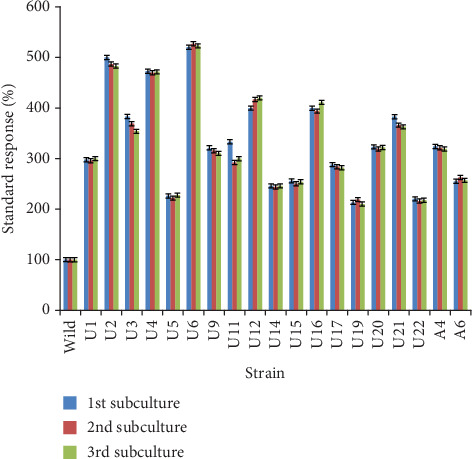
Standard response of the wild and mutant strains in successive subcultures.

A total of 34 mutants (10 mutants exposed to nitric acid and 24 exposed to UV rays) were selected to evaluate their ability to produce glucose oxidase. Hyperproducing GOD mutants, known as positive mutants, were secondarily selected on the basis of the brownish-red diffusion zone on agar plates. The obtained results (Figure 2) indicated that most mutant strains showed a better performance compared to the parent strain. According to the data, the efficiency of mutant strains to produce GOD depended on the type of mutation treatment. Indeed, mutant strains treated by UV radiation showed a significant improvement of GOD production compared to the other mutant strains treated by nitric acid. It has been reported in the literature [25, 26] that physical mutagenesis is a cost-effective method to generate potential mutant-derived strains that may be used for commercial production of the enzymes. Ghani et al. [27] reported that UV rays excite electrons in the molecule, as a result of which extra bonds are formed between adjacent pyrimidines. This alteration can be repaired by the DNA repair mechanism, but sometimes, it may lead to the mutation. Doudney and Young [28] suggested that ultraviolet rays are destructive but they have the capability to generate mutants with enhanced performance and ability for better environmental adaptation. Among the hyperproducing GOD strains, nineteen mutants demonstrated the largest and most intense brown zones and showed the greatest standard response larger than 200%. In several microbial mutagenesis reports, UV treatment was usually followed by a chemical treatment to avoid back mutation [29]. However, the finding showed that these mutagens were stable without any drastic changes in the GOD production for three successive subcultures indicating that the developed mutants were highly stable (Figure 3).

In order to identify accurately the overproducing GOD mutants, the selected mutants were then cultivated subsequently at the optimal culture conditions in submerged fermentation [11]. The obtained results (Table 1) confirm a great predisposition of the selected mutants to produce GOD. In comparison with the starting strain *A*. *tubingensis* CTM 507, a significant increase in GOD activity (*P* < 0.05) was obtained from 48.93 to over 218.03%. It was clear from the result that the maximum GOD activity was produced by mutant strains treated by UV irradiation. Indeed, mutant strains U4, U11, U12, U16, U20, and U21 present an important improvement in GOD production. Accordingly, it provided, respectively, the GOD productivity of 5186, 5426, 6051, 6903, and 6411 (U/g of substrate). Similar results were observed by Zia et al. [26] who studied the effect of gamma irradiation on *Aspergillus niger* for enhanced production of glucose oxidase. They reported a significant increase in enzyme activity (between 274 and 366.6%) after treatment. Also, Khattab and Bazaraa [30] noted an important increment in GOD production of 393.8% after UV treatment of *A*. *niger*.

### 3.3. Insecticidal Activity of GOD against *E*. *kuehniella* Larvae

**Figure 4:**
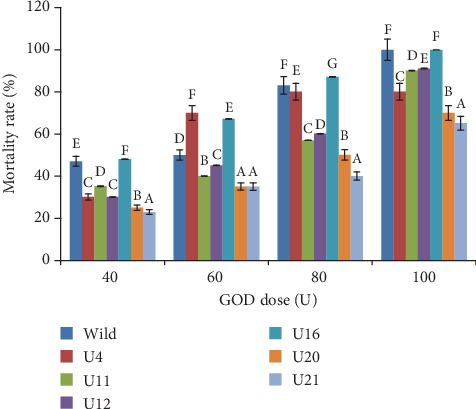
Mortality rate of *Ephestia kuehniella* exposed to different doses of GOD from the wild and mutant strains. Statistical difference is shown by different letters (*P* < 0.05) between strains.

The bioassay of GOD produced by the wild strain *A*. *tubingensis* and the selected mutant strains (U4, U11, U12, U16, U20, and U21) was performed against the first instar larvae of *E*. *kuehniella*. The findings revealed that partially purified GOD produced by the wild and mutant strains was toxic to *E*. *kuehniella*. The insecticidal activity of the GOD produced by both the wild and mutant strains was dose dependent. Indeed, a gradual increase of insect rate mortality was observed by increasing the GOD dose ranging from 0 to 100 U (Figure 4). After five days of exposure, the wild and mutant GOD concentrations of 80 U caused mortality rate to be about 50%. GOD concentration of 100 U produced by mutant strains U4, U16, and U21 induced 100% mortality.

The investigation of the effect of the mutant's GOD enzyme on the *E*. *kuehniella* revealed an improvement of the toxicity. Indeed, bioassays demonstrated that the majority of mutant strains exhibited an enhancement in their insecticidal activity when compared to the wild strain. The comparison of the LC_50_ and LC_90_ values of GOD secreted by the wild and mutant strains showed that some of the GOD mutants were improved in their efficiency against the first instar larvae of *E*. *kuehniella* (Table 2). The enhancement of the GOD efficiency was detected in the case of mutants U11, U12, U20, and U21. These results were in agreement with the studies of Hmani et al. [31], who investigated the improvement of Vip3 toxin production and its efficiency through classical mutagenesis of *B*. *thuringiensis*, showed that some of the mutants were highly efficient in their Vip3 toxins against *Spodoptera littoralis* with lower values in their lethal concentrations, and demonstrated that this improvement was independent of the kind of treatments, nitrous acid or UV rays.

It was largely reported in the literature that the GOD is widely used as an antimicrobial agent due to its ability to inhibit the growth of microbes by naturally produced hydrogen peroxide [32]. Kriaa et al. [11, 12] studied the antifungal activity of GOD from *Aspergillus tubingensis* CTM 507 against different phytopathogenic fungi. They reported that the partially purified GOD has a potential antifungal activity against *Fusarium solani* [11] and *Pythium ultimum* [12]. In fact, GOD (125 AU) inhibited *Fusarium solani* growth and spore production and caused the reduction of spores, the formation of chlamydospores, the induction of mycelial cords, and the vacuolization of mycelium [11]. At a concentration of 30 AU, the enzyme generated a strong swelling and deformation of *P*. *ultimum* mycelia and a significant lethal damage of the fungi [12]. Recently, Li et al. [33] showed that GOD destroyed spore cell membranes and structures of *Botrytis cinerea* and inhibited its spore germination and germ tube elongation. They demonstrated that the production of gluconic acid and hydrogen peroxide led to the growth inhibition of *B*. *cinerea*. However, the action mode of GOD inside the insect remains unknown and is not well documented. To the best of our knowledge, there were no earlier reports regarding the insecticidal activity of the microbial GOD.

Interestingly, several studies have focused their attention on the use of glucose oxidase, from the labial gland of herbivore insect, to counteract insect pathogens and plant defenses. Through its catalytic product, GOD suppressed infectivity of potential pathogens [13, 14]. Basu et al. [34] mentioned that GOD mediated suppression of defenses in tobacco. Similarly, Musser et al. [35] found that glucose oxidase was the principal salivary enzyme responsible for suppressing the induction of nicotine in wounded tobacco plants. Louis et al. [16] demonstrated that GOD from *H*. *zea* saliva and *Ostrinia nubilalis* induced the direct and indirect defenses in tomato. Recent reports noted that GOD importantly inhibit plant defenses by attenuating the ethylene and jasmonic acid levels and eliciting the salicylic acid burst [14, 15].

### 3.4. Histopathological Effect of GOD in *E*. *kuehniella* Midgut Larvae

**Figure 5:**
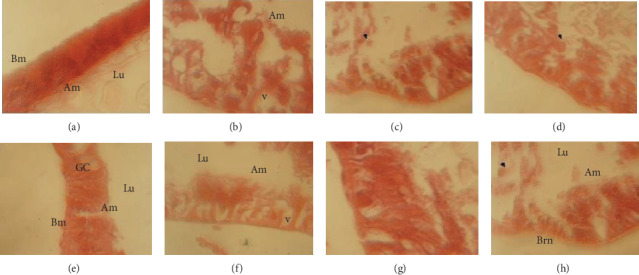
Histopathological effects of glucose oxidase on *E*. *kuehniella* midguts. (a) Section through the midgut epithelium of control larvae not exposed to toxins. (b) Section through the midgut epithelium of *E*. *kuehniella* larvae fed with wild strain-containing diet. (c) Section through the midgut epithelium of *E*. *kuehniella* larvae fed with U4-containing diet. (d) Section through the midgut epithelium of *E*. *kuehniella* larvae fed with U11-containing diet. (e) Section through the midgut epithelium of *E*. *kuehniella* larvae fed with U12-containing diet. (f) Section through the midgut epithelium of *E*. *kuehniella* larvae fed with U16-containing diet. (g) Section through the midgut epithelium of *E*. *kuehniella* larvae fed with U20-containing diet. (h) Section through the midgut epithelium of *E*. *kuehniella* larvae fed with U21-containing diet. (a) Regular epithelial cell and well-developed lumen contents. (b–h) Extensive damage of the midgut epithelium. Lu: lumen; Am: apical membrane; Bm: basal membrane; V: vacuole formation. Magnification: 40x.

The histopathological changes occurring in the larval midgut of the third instars of *E*. *kuehniella* treated and not treated with partially purified GOD were investigated. The midgut section of the untreated larvae showed the preserved layer of epithelial cells with regular placed microvilli bordering the midgut lumen (Figure 5(a)). Midgut larvae treated with GOD produced by the wild and the selected mutant strains (U4, U11, U12, U16, U20, and U21) exhibited structural changes that included altered shape, disruption of the basement membrane, cytoplasm vacuolization, and appearance of vesicles at the apical part of the cells toward the midgut lumen (Figures 5(b), 5(d)–5(h)). The histopathological effects observed with the partially purified enzyme were similar to those of *B*. *thuringiensis* Vip3Aa16 [36], *B*. *subtilis* biosurfactant [10], and *B*. *thuringiensis* Vip3 (459) [37] in the *E*. *kuehniella* midgut.

## 4. Conclusion

The finding reported the improvement of glucose oxidase secreted by the *A*. *tubingensis* strain through random mutagenesis. The obtained results revealed that treatment of *E*. *kuehniella* with partially purified GOD could offer an effective control of *E*. *kuehniella* larvae. It also clearly demonstrates that proteolysis of this enzyme in the larval midgut could be a key step in determining their potency against different susceptible pests. The data encourages the use of the selected mutants to produce important quantities of this pest control agent and the possibility of its use for the formulation of new bioinsecticides.

## Figures and Tables

**Table 1:** GOD production by different selected mutants in submerged fermentation.

Strain	Productivity (U/g of substrate)	Amelioration	Deterioration
Wild	2797 ± 0.28^g^	0	0
U1	5150 ± 0.04^j^	84	0
U2	6879 ± 0.05^o^	145	0
U3	8896 ± 0.03^r^	218	0
U4	5186 ± 0.020^k^	85	0
U5	588 ± 0.02^a^	0	79
U6	2425 ± 0.02^f^	0	13
U9	4166 ± 0.01^i^	49	0
U11	5426 ± 0.02^l^	94	0
U12	6051 ± 0.01^m^	116	0
U14	1128 ± 0.02^c^	0	59
U15	1945 ± 0.03^e^	0	30
U16	6903 ± 0.03^p^	146	0
U17	648 ± 0.02^b^	0	77
U20	7672 ± 0.02^q^	174	0
U21	6411 ± 0.03^n^	129	0
A4	1909 ± 0.03^d^	0	31
A6	2941 ± 0.03^h^	5	0

Mean ± standard error. Different letters in the same column are significantly different (*P* < 0.05) between strains.

**Table 2:** Lethal concentrations LC_50_ and LC_90_ of GOD produced by wild and mutant strains on the first instar of *E. kuehniella* larvae.

Strain	LC_50_ (U/cm^2^)	LC_90_ (U/cm^2^)
Wild	61.70 ± 6.86^c^	86.37 ± 8.07^ab^
U4	58.54 ± 17.95^a^	118.76 ± 30.97^ab^
U11	89.01 ± 13.70^f^	154.63 ± 56.19^b^
U12	88.51 ± 13.73^f^	141.61 ± 47.68^ab^
U16	59.83 ± 7.40^b^	84.622 ± 8.03^a^
U20	80 ± 9.01^d^	128.87 ± 28.97^ab^
U21	86.24 ± 10.41^e^	139.69 ± 37.44^ab^

Mean ± standard error. Different letters in the same column are significantly different (*P* < 0.05) between strains.

## Data Availability

No data were used to support this study.
